# Synthesis of Novel Analogs of Thieno[2,3-*d*] Pyrimidin-4(3*H*)-ones as Selective Inhibitors of Cancer Cell Growth

**DOI:** 10.3390/biom9100631

**Published:** 2019-10-21

**Authors:** Sheng Zhang, Feize Liu, Xueling Hou, Jianguo Cao, Xiling Dai, Junjie Yu, Guozheng Huang

**Affiliations:** 1 College of Life Sciences, Shanghai Normal University, Shanghai 201418, China; zs534321312@foxmail.com (S.Z.); daixiling2010@shnu.edu.cn (X.D.); 13621600677@163.com (J.Y.); 2 Key Laboratory of Plant Resources and Chemistry of Arid Zone, Xinjiang Technical Institute of Physics and Chemistry, Chinese Academy of Sciences, Urumqi 830011, Chinaxlhou@ms.xjb.ac.cn (X.H.); 3 University of Chinese Academy of Sciences, Beijing 100049, China

**Keywords:** thienopyrimidine, thieno[2,3-*d*] pyrimidinone, anticancer

## Abstract

New 2,3-disubstituted thieno[2,3-*d*]pyrimidin-4(3*H*)-ones were synthesized via a one-pot reaction from 2*H*-thieno[2,3-*d*] [1,3]oxazine-2,4(1*H*)-diones, aromatic aldehydes, and benzylamine or 4-hydroxylbezylamine. The obtained compounds were tested in vitro for cancer cell growth inhibition. Compound **19** can inhibit all four types of tested cancer cells, i.e., MCF-7, A549, PC-9, and PC-3 cells. Most of the compounds inhibited the proliferation of A549 and MCF-7 cells. Compound **15** exhibited the strongest anti-proliferative effect against A549 cell lines with IC_50_ values of 0.94 μM, and with no toxicity to normal human liver cells. Its potency was further proved by cell clone formation assay, Hoechst 33258 staining, and evaluation on the effects of apoptosis-related proteins.

## 1. Introduction

Cancer is a broad term that describes a group of more than 200 diseases which endow the characteristics of uncontrolled growth and division of cells. Eventually, they can form into tumors, impair the immune system, and subsequently prevent normal body function [1,2]. The complexity of cancer renders it the leading cause of global mortality. Statistical data showed that there were 14.1 million new cases and 8.2 million deaths in 2012 [2]. In 2018, the data jumped to 18.1 million new cancer cases and 9.6 million cancer deaths [3].

Among all the types of cancer, lung cancer is the most commonly diagnosed and the leading cause of cancer death in both men and women. Lung cancer can usually be divided into small cell lung cancer and non-small cell lung cancer (NSCLC), and most lung cancer cases (~90%) are NSCLC [4,5]. In general, about half of NSCLC cases are diagnosed in the advanced stage [6]. Although in recent years, significant progress has been made in surgery, radiotherapy, chemotherapy, and targeted therapy, the five-year survival rate of patients with advanced NSCLC is still relatively low [7]. For patients who are not healthy enough for surgery, chemotherapy is the main approach for the treatment of NSCLC. Meanwhile, dozens of drugs for the treatment of NSCLC have been developed, including gefitinib, erlotinib, cetuximab, and bevacizumab, etc. FDA has approved more than 60 drugs or drug combinations to be used to treat non-small cell lung cancer. Unfortunately, the effectiveness and expectancy of life are still not satisfactory for most of the drugs. In addition, the price of many clinically used drugs is over expensive for impoverished patients in undeveloped and developing countries. For example, crizotinib is sold at the price of 1.56 thousand yuan/60 tablets in China, which is unaffordable to the majority of patients there. Therefore, the search for new types of drugs for the treatment of cancer, especially lung cancer, is still a great emergency [8,9].

**Figure 1:**
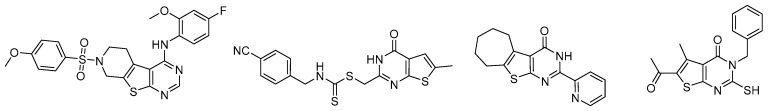
Recently reported representative compounds with the thieno[2,3-*d*] pyrimidine motif as potent anti-cancer agents. [20,29,30,31].

Thieno[2,3-*d*]pyrimidines are a type of pyrimidine structurally fused with thiophene [10]. They can be regarded as bioisosteres of quinazolines and nucleobases which possibly possess similar biological activities as such [11,12]. In fact, thieno[2,3-*d*]pyrimidines with different substituted groups have been prepared and evaluated with regard to pharmaceutical functions, such as 17 beta-hydroxysteroid dehydrogenase inhibition, SIRT2 inhibition, and antimycobacterial and anti-cancer agent effects (Figure 1), etc. [13,14,15,16,17,18,19,20].

Therefore, investigation for the preparation of thieno[2,3-d]pyrimidin-4(3*H*)-ones has intrigued researchers to make numerous efforts in synthetic methodology [21,22,23,24,25]. In the course of searching for new cholinesterase inhibitors based on evodiamine, we reported a new one-pot protocol for the straightforward synthesis of rutaecarpine and its analogs from isatoic anhydride and 4,9-dihydro-3*H*-pyrido[3,4-*b*]indole [26,27]. Noticing the structural similarity between isatoic anhydride and 2*H*-thieno[2,3-*d*][1,3]oxazine-2,4(1*H*)-dione, a convenient one-pot synthesis of 2,3-disubstituted thieno[2,3-*d*]pyrimidin-4(3*H*)-ones was reported by us [28]. This method can provide the target compounds easily and free of tedious chromatographical purification and encourage the preparation of more compounds, and subsequently, to evaluate their activities as possible anti-cancer agents.

## 2. Materials and Methods

### 2.1. Synthesis of 2,3-Disubstituted Thieno[2,3-d] Pyrimidin-4(3H)-Ones

**Scheme 1:**
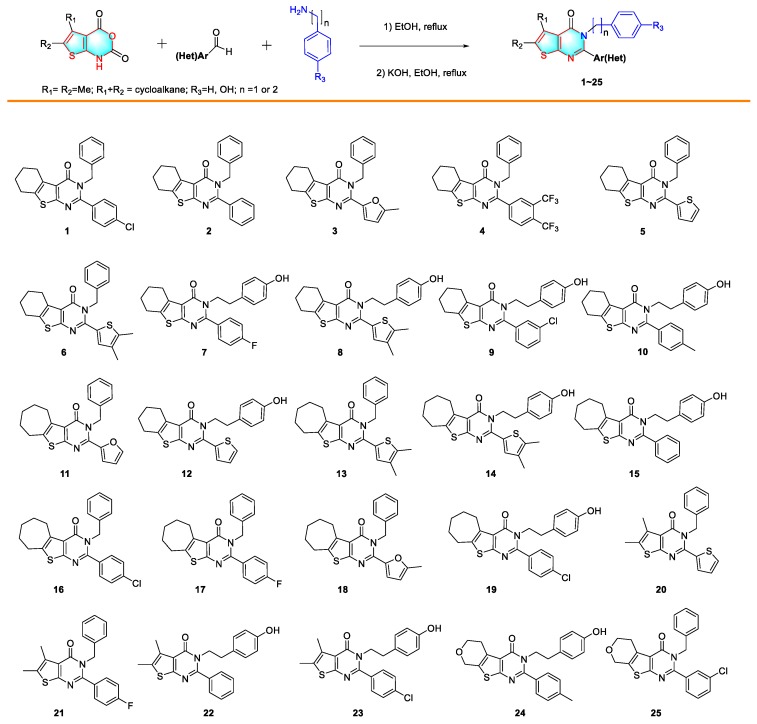
Synthesis of 2,3-disubstituted thieno[2,3-*d*]pyrimidin-4(3*H*)-ones.

The method of synthesis of 2,3-disubstituted thieno[2,3-*d*]pyrimidin-4(3*H*)-ones was reported previously [28], as shown in Scheme 1. The four substituted 2*H*-thieno[2,3-*d*] [1,3] oxazine-2,4(1*H*)-diones, including 5,6,7,8-tetrahydro-2H-benzo [4,5] thieno[2,3-*d*] [1,3]oxazine-2,4(1*H*)-dione; 1,5,6,7,8,9-hexahydro-2*H*,4*H*-cyclohepta[4,5]thieno[2,3-*d*] [1,3]oxazine-2,4-dione; 5,6-dimethyl-2*H*-thieno[2,3-*d*] [1,3] oxazine-2,4(1*H*)-dione; and 1,5,6,8-tetrahydro-2*H*,4*H*-pyrano[4′,3′:4,5]thieno[2,3-*d*] [1,3]oxazine-2,4-dione, were synthesized in a three-step scheme [28]. The obtained 2*H*-thieno[2,3-*d*] [1,3] oxazine-2,4(1*H*)-dione was then suspended in ethanol, and mixed with respectful aromatic aldehyde, and benzylamine or 4-hydroxybenzylamine. The mixture was heated for 10–14 h before the addition of potassium hydroxide. Additional stirring while heating for 6–10 h furnished the target compounds in good to high yields (46%–86%). The spectrum of nuclear magnetic resonance (NMR) and high-resolution mass spectrometry (HRMS) analyses are consistent with the structures. Detailed characterization data by ^1^H, ^13^C NMR, and HRMS for newly synthesized compounds are available in Supplementary Materials associated with this article.

### 2.2. Cell Culture and Reagents

Primary human liver HL-7702 cells were purchased from SIBS (Shanghai Institutes for Biological Sciences, Shanghai, China) while MCF-7, A549, PC-9 and PC-3 cells were from ATCC (American Type Culture Collection, Rockville, MD, USA). HL-7702, PC-9 and A549 cells were incubated with Roswell Park Memorial Institute (RPMI) 1640 medium and MCF-7 were cultured in Dulbecco’s Modified Eagle’s Medium (DMEM) supplemented with 10% heat-inactivated fetal bovine serum (FBS) and 1% antibiotics (penicillin and streptomycin). PC-3, a human prostate adenocarcinoma cell, was maintained in Ham’s F-12 Medium supplemented with 10% FBS, 1% penicillin/streptomycin and 1% glutamine. All cells were cultured at 37 °C in an incubator containing 5% CO_2_.

### 2.3. MTT (Methyl Thiazolyl Tetrazolium Bromide) Assay

The cell viability was determined by using the MTT (methyl thiazolyl tetrazolium bromide) assay. Briefly, cells were seeded in 96-well plates (4 × 10^3^ cells/well) overnight and exposed to the synthesized compounds with various concentrations. Camptothecin and dimethyl sulfoxide (DMSO) served as a positive control and a negative control respectively. After 72 h of incubation, 20 μL of MTT (5 mg/mL in phosphate buffer saline (PBS)) were added into each well. Dissolved yellowish MTT is converted to an insoluble purple formazan during incubation (4 h). In order to dissolve the formazan crystals, 100 μL of lysis solution (10% SDS (sodium dodecyl sulfate)-5% isobutyl alcohol-0.01M HCl) was added into per well. Finally, the optical density (OD) of solution in the plates was measured using a microplate reader at 570 nm after dissolving overnight. Cell viability was calculated by using the following formula: (OD_control_ − OD_treated_)/OD_control_ × 100%.

### 2.4. Colony Formation Assays

The colony formation assay was executed to assess the colony forming ability of cancer cells treated by compounds. After being trypsinized and counted, the cells were seeded again on a 6-well plate with 4000 cells/well. Subsequently, cells were incubated at 37 °C in 5% CO_2_ overnight with medium. Afterwards, the cells were being treated with drugs (1 μM, 10 μM) and camptothecin (0.1 μM) for 10 days. Then, fresh PBS (1 mL/well) was added to wash the cells twice, before fixing with the stationary solution (10% acetic acid- 10% methanol- 80% dd H_2_O, 1 mL/well) at room temperature for 30 min. After that, 1% crystal violet was used to stain the cells. The cell viability was evaluated by the number of purple colonies.

### 2.5. Hoechst 33258 Staining

For Hoechst staining, A549 cells were seeded in 6-well plates (8 × 10^4^ cells/well) and allowed to adhere overnight. Compounds at a final concentration of 10, 5 and 1 µM were used to treat cells in the exponential growth phase for 72 h. Following this, the staining solution was added to the well plates and held at room temperature for 10 min. Afterwards, Hoechst 33258 was added to dye the cells for 10 min at room temperature in the dark. After washing two times with PBS, the treated cells were photographed under a semi-electrical fluorescence microscope (Olympus BX53, Olympus, Tokyo, Japan), to capture images of the stained nuclei from each well from randomly selected regions. If the nuclei showed chromatin condensation and fragmentation, the cells were considered to be apoptotic.

### 2.6. Caspase-3 Activity Assay

Briefly, A549 cells were cultured at 2 × 10^5^ cells/well in 6-well plates and incubated with different concentrations of compounds (0, 1, 5 µM) for 48 h. At the end of the treatment, cells were harvested and lysed by the addition of lysis buffer from the caspase-3 assay kit. Aliquots of cell lysate were added to 96-well plates and the supernatant of cell lysates were mixed with buffer containing the above substrate peptides for 2 h at 37 °C. The absorbance in each well was measured at 405 nm using a microplate reader.

### 2.7. Western Blotting

Total protein samples were extracted from A549 cells. cells were treated with different concentrations of compounds for 72 h, then the cells were collected, homogenized in 100 µL of ice-cold lysis buffer containing 2% SDS (sodium dodecyl sulfate), 10% DTT (dithiothreitol), 10% glycerin, 0.1% BPB (bromophenol blue) and 50mM Tris buffer (pH 6.8). After cell lyses were boiled for 30 min at 100 °C, samples were resolved on 12% SDS-PAGE (polyacrylamide gel) and then transferred to nitrocellulose (NC) membranes. Then the membranes were blocked with 5% skim milk at room temperature for 1 h. After incubation, the membranes were probed by primary antibodies for β-actin, Bax (BCL2-associated X protein) and Bcl-2 (B-cell lymphoma-2) at 4 °C overnight. After the blots were washed in TBST (Tris-base solution-Tween 20), membranes were incubated with HRP (horseradish peroxidase) -conjugated IgG secondary antibodies for 1 h at 37 °C. Finally, a Chemi-Doc image analyzer was used to determine protein expression levels by analyzing the signals captured on the NC membranes.

### 2.8. Statistical Analysis

The GraphPad Prism 5.0 software program (GraphPad Software, La Jolla, USA) was used to determine the statistical significance of differences between the experimental and control groups. Analyses were performed in triplicate and the results presented as means ± SD (standard deviation). Significance threshold was fixed at * *p* < 0.05, ** *p* < 0.01, *** *p* < 0.001.

## 3. Results and Discussion

### 3.1. Cytotoxicities of Synthesized 2,3-Disubstituted Thieno[2,3-d] Pyrimidin-4(3H)-Ones

As compounds containing thieno[2,3-d]pyrimidine motif have been reported to possess cytotoxicity against cancer cell lines [20,29,30,31], we evaluated the antiproliferative activity of thieno[2,3-d]pyrimidin-4(3H)-one compounds synthesized herein, against several human cancer cells, as well as human normal liver cells (HL-7702). The cancer cells used in the research include human non-small cell lung cancer cells A549, PC-9, human prostate cancer cell PC-3, and human breast cancer cell line MCF-7. Topoisomerase I inhibitor camptothecin was used as a positive control compound [32].

The results summarized in Table 1 showed that most of the synthesized compounds are not toxic to normal human liver cells with IC_50_ values higher than 100 μM (except for compound **3**, **14** and **19**), indicating that these compounds are possibly safe for further development. The majority of synthesized compounds are not toxic to PC-3 and PC-9 cells with IC_50_ values higher than 100 μM, with only a few exceptions. On the contrary, most of the compounds inhibit the proliferation of A549 and MCF-7 cells. The cytotoxities of compounds **4**, **7**, **10**, **19**, and **22** against MCF-7 are satisfactory. In addition, compounds **7**, **10**, and **19** are also potently against A549 with IC_50_ values less than 10 μM. Compound **19** is the only one inhibiting all four types of tested cancer cells. Taking the chemical structure into consideration, we believe that the 4-hydroxylbenzy-substituted compounds are more favorable than others. Among all the synthesized 25 compounds, compound **15** is the most effective, as it inhibits the proliferation of A549 with IC_50_ values of 0.94 μM. Therefore, we further evaluated compound **15** in the following assays.

### 3.2. Cell Clone Formation Assay of Compounds ***15***

**Figure 2:**
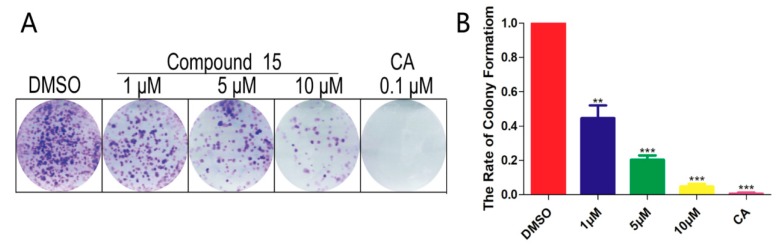
Compound **15** was shown to inhibit the colony formation of cancer cell lines. (**A**) The colony formation of A549 cells could be reduced by compound **15** in a dose dependent manner. Compound **15** incubated with cancer cells for 7 days. (**B**) Quantification of the rate of colony formation described in A. Statistically significant differences are presented as * *p* < 0.05, ** *p* < 0.01, *** *p* < 0.001, compared with the control group. CA: Camptothecin.

To explore the significant anti-tumor activity of A549, we used a colony test to demonstrate whether compound **15** can inhibit the proliferation of A549 cells. Similar to what we observed in the MTT assay, the inhibition rate was 56% at a low concentration (1 μM), indicating that compound **15** could dose-dependently inhibit the proliferation of cancer cells (Figure 2).

### 3.3. Apoptosis Analysis of Compound ***15*** by Hoechst 33258 Staining

**Figure 3:**
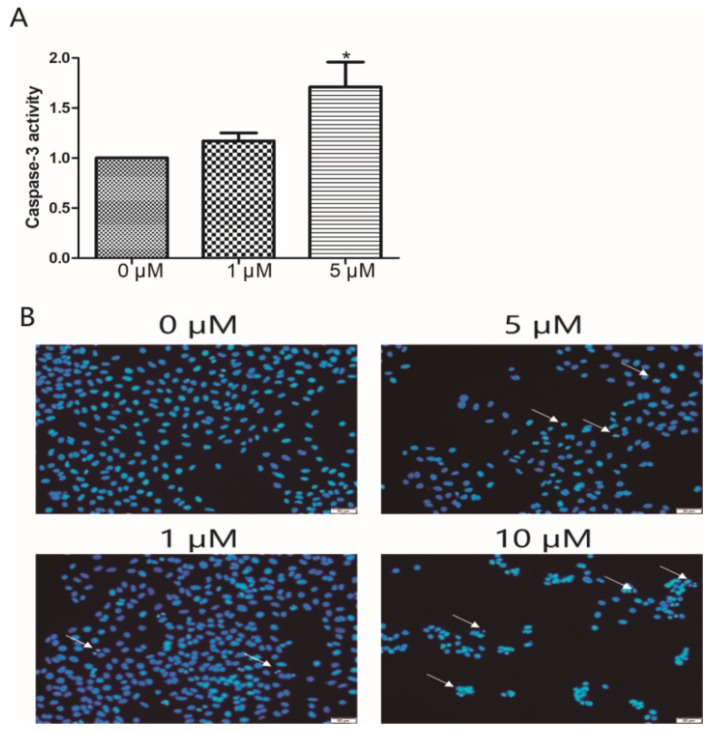
Compound **15** treatment induces A549 cell apoptosis. (**A**) Activation of caspase-3 by compound **15** (0, 1, 5 μM). (**B**) Morphology of nuclei in A549 cells treated with compound **15** (0, 1, 5, 10 μM), stained by Hoechst 33258, and observed under a fluorescence microscope. The white arrows represent apoptotic cells. Data are expressed as means standard deviation from three independent experiments. * *p* < 0.05, ** *p* < 0.01 compared with the control group.

To determine the potential of compounds to induce apoptosis, the Hoechst 33258 staining technique was used to study the changes in apoptosis after compounding A549 cells. As shown in Figure 3, the nuclei in the control A549 cells were uniformly stained without significant morphological changes. Cells treated with compound **15** (1 μM) resulted in brightly stained nuclei due to chromatin condensation (indication of apoptosis); and when the dose was increased to 5 and 10 μM, cell membrane rupture and contraction were observed. Meanwhile, chromatin displayed concentration, fragmentation, and small nuclei, and the number of cells was significantly reduced. These results revealed that apoptosis is involved in the toxicity of compound **15** to A549 cells.

### 3.4. Effects on Apoptosis-Related Proteins of Compound ***15***

In the process of apoptosis, the Bcl-2 protein family is often studied as it has important regulatory effects. It is accepted that the key factor for regulation is the ratio of Bcl-2 to Bax [33]. Simultaneously, Bcl-2 and Bax can not only act as the upstream regulation mechanism of caspase-3 participating in the adjustment of its activity [34], but also can act as a direct substrate of caspase-3 to the downstream. They are related to each other in the process of apoptosis conduction. Meanwhile, caspase-3, which acts as an “apoptotic executor”, activates and cleaves PARP (Poly ADP-ribose polymerase), etc., and thereby affects DNA replication, transport, damage repair, and induction of apoptosis [35].

**Figure 4:**
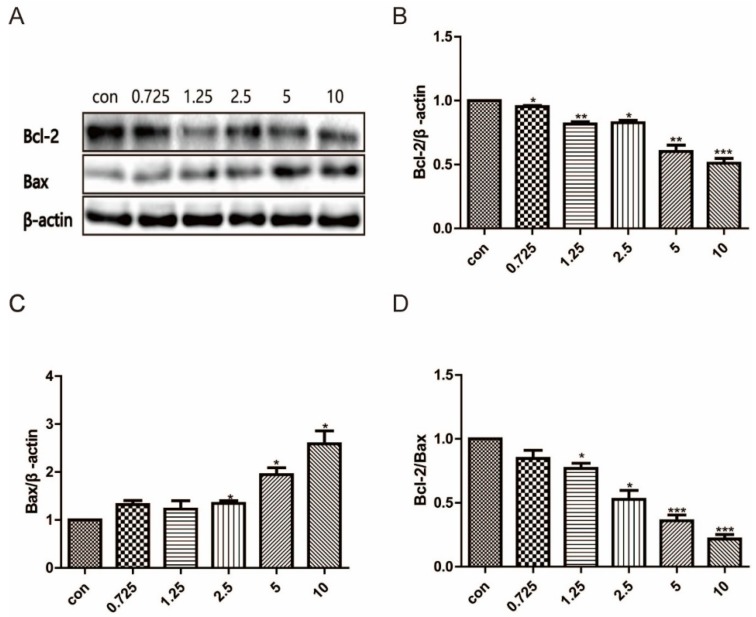
A549 cells were treated with compound **15** (0, 0.725, 1.25, 2.5, 5, 10 μM) for 72 h. Protein expression levels of Bcl-2 and Bax were measured using western blot analysis. (**A**) Protein expression levels of Bcl-2 and Bax were measured using western blot analysis. Representative western blot images are shown. (**B**) Grayscale value analysis of Bcl-2, (**C**) Bax and (**D**) Bcl-2/Bax ratio. Data are expressed as means standard deviation from three independent experiments. * *p* < 0.05, ** *p* < 0.01 compared with the control group.

Several studies have reported that Bcl-2, Bax, and caspase-3 are pivotal proteins that cause apoptosis in lung cancer cells [36]. To demonstrate the regulatory roles of compound **15** in apoptosis, we analyzed the expression changes of Bcl-2 and Bax by western blotting and the enzyme activity of caspase-3. As shown in Figure 4, in comparison with the control cells, compound **15** induced an increase of Bax level and a reduction of Bcl-2 level in a dose-dependent model. Thus, compound **15** caused a significant decrease in the proportion of Bcl-2/Bax compared to the control in A549 cells. Treatment with compound **15** caused an accumulation of caspase-3 in the cytosol, most probably due to activation of apoptosis. These results suggest that compound **15** downgrades the Bcl-2/Bax ratio, and leads to the release of caspase-3 activation, which triggers the execution of apoptosis.

## 4. Conclusions

In summary, a series of novel derivatives of thienopyrimidinone were obtained using a one-pot, two-step, three-component method. All the synthesized compounds were evaluated for their cytotoxic effects against A549, PC-3, MCF-7, PC-9, and HL-7702 cell lines. Although most of these compounds are not toxic to PC-3 and PC-9 cells, they can inhibit the proliferation of A549 and MCF-7 cells. Compounds **7**, **10**, and **19** are potently against MCF-7 and A549. Compound **19** can inhibit all four types of cancer cells that we tested. Compound **15** exhibited the strongest anti-proliferative potency against A549 cell lines with IC_50_ values of 0.94 μM, and no toxicity to normal human liver cells. Further mechanistic studies indicate that compound **15** possesses a significant inhibitory effect on the community formation ability of A549. Nuclear staining experiments showed that compound **15** induced nuclear agglomeration and fragmentation of A549 cells, which promoted apoptosis. Western blot and enzymatic activity experiments further confirmed that compound **15** further activated caspase-3 activity by up-regulating Bax and decreasing the level of Bcl-2 expression, thereby inducing apoptosis of lung cancer cells. Taken together, compound **15** deserves further investigation as a potential chemotherapeutic agent for lung cancer.

## Figures and Tables

**Table 1:** Cytotoxic activity * (IC_50_, μM) of compounds **1**–**25**.

Compound	Cell Lines				
	HL-7702	PC-3	PC-9	MCF-7	A549
**1**	>100	>100	>100	>100	>100
**2**	>100	>100	>100	>100	>100
**3**	55.17 ± 0.50	92.93 ± 2.29	86.09 ± 1.28	>100	54.42 ± 1.83
**4**	>100	>100	>100	24.67 ± 2.04	58.24 ± 0.65
**5**	>100	>100	80.97 ± 3.94	97.50 ± 0.13	>100
**6**	>100	>100	>100	59.86 ± 0.34	17.38 ± 0.88
**7**	>100	>100	44.76 ± 4.47	10.36 ± 1.32	6.86 ± 0.98
**8**	>100	87.75 ± 0.63	92.97 ± 0.52	>100	50.92 ± 0.07
**9**	>100	>100	>100	30.70 ± 0.29	19.59 ± 0.33
**10**	>100	43.43 ± 2.73	49.68 ± 2.27	27.47 ± 0.02	8.51 ± 0.16
**11**	>100	>100	>100	65.63 ± 0.26	51.42 ± 0.46
**12**	>100	21.95 ± 1.76	54.20 ± 2.06	36.39 ± 1.41	11.65 ± 0.12
**13**	>100	92.52 ± 1.89	>100	75.94 ± 0.17	49.86 ± 0.30
**14**	68.64 ± 0.26	84.68 ± 0.48	86.33 ± 0.22	46.09 ± 0.48	49.83 ± 0.68
**15**	>100	47.51 ± 3.39	>100	17.32 ± 0.20	0.94 ± 0.01
**16**	>100	>100	>100	>100	80.96 ± 1.04
**17**	>100	>100	>100	>100	>100
**18**	>100	92.03 ± 2.34	58.28 ± 0.18	96.36 ± 0.72	63.20 ± 0.03
**19**	19.16 ± 0.06	25.14 ± 0.37	26.71 ± 1.38	13.04 ± 0.11	6.26 ± 0.87
**20**	>100	>100	>100	>100	80.09 ± 0.83
**21**	>100	>100	>100	>100	>100
**22**	>100	>100	>100	21.89 ± 0.82	>100
**23**	>100	>100	>100	70.64 ± 0.59	46.14 ± 0.17
**24**	>100	>100	>100	>100	32.92 ± 1.84
**25**	>100	94.21 ± 1.83	>100	>100	43.95 ± 0.71
**CAMPTOTHECIN**	0.11 ± 0.03	0.85 ± 0.01	0.09 ± 0.01	0.26 ± 0.05	0.10 ± 0.02

*,^a^ IC_50_ = compound concentration required to inhibit tumor cell proliferation by 50%. Data are expressed as the mean ± standard error from the dose–response curves of at least three independent experiments.

## Supplementary Materials

Supplementary materials are available online at https://www.mdpi.com/2218-273X/9/10/631/s1.
