# Supplementary material for: Synthesis of Novel Analogs of Thieno[2,3-d] Pyrimidin-4(3H)-ones as Selective Inhibitors of Cancer Cell Growth
Source: Biomolecules. 2019 Oct 21;9(10):631. doi: 10.3390/biom9100631 (PMC6843832; doi:10.3390/biom9100631)
Supplement: Supplementary file 1 [file biomolecules-09-00631-s001.pdf]

Supporting Material

for

Synthesis of novel analogs of thieno[2,3-d] pyrimidin-4(3H)-ones as selective inhibitors of cancer cell growth

Sheng Zhang [a], Feize Liu [b, c], Xueling Hou [b], Jianguo Cao [a, *], Xiling Dai [a], Junjie Yu [a], Guozheng Huang [a,b,*]

[a] College of Life Sciences, Shanghai Normal University, Shanghai, 201418, P. R. China

[b] Key Laboratory of Plant Resources and Chemistry of Arid Zone, Xinjiang Technical Institute of Physics and Chemistry, Chinese Academy of Sciences, Urumqi, 830011, P. R. China.

[c] University of Chinese Academy of Sciences, Yuquan Rd 19 A, Beijing, 100049, P. R. China

⁎ Corresponding author: cao101@shnu.edu.cn (J. Cao); guozheng.huang@yahoo.com, g.huang@ms.xjb.ac.cn (G. Huang);

Synthesis

The general procedure for synthesis of the 2,3-disubstittued thieno[2,3-*d*]pyrimidin-4(3*H*)-ones was published previously [*SynOpen* **2018**, *02*, 207]. The compounds were purified by column chromatography over silica gel (Qingdao Haiyang Chemical Co., 200–300 mesh). Melting points were determined on a Buchi B-540 apparatus and uncorrected. NMR spectra were recorded with a Varian 400 MHz or a 600MHz NMR spectrometer in $CDCl_3$ or DMSO-d_6, using TMS as an internal standard. High-resolution mass spectra (HRMS) were recorded on UHPLC-Q-Orbitrap-MS. The purities of compounds were confirmed by HPLC (Ulitmate 3000, Thermo Scientific) to be over than 95%. Analytic data of compounds **4**, **5**, **9**, **11**, **14**, **16**, **17**, **20**, and **22** were presented below, other compounds was reported previously [*SynOpen* **2018**, *02*, 207].

3-benzyl-2-(3,4-bis(trifluoromethyl)phenyl)-5,6,7,8-tetrahydrobenzo[4,5]thieno[2,3-d]pyrimidin-4(3H)-one (**compound 4**)

Yield 56%, white solid; m.p. 161-163 °C; ^{1}H NMR (400 MHz, $CDCl_3$) δ7.93 (s, 1H), 7.66 (s, 2H), 7.25 – 7.20 (m, 3H), 6.88 – 6.80 (m, 2H), 5.19 (s, 2H), 3.12 (t, *J* = 5.8 Hz, 2H), 2.84 (t, *J* = 4.8 Hz, 2H), 1.99-1.95 (m, 4H). ^{13}C NMR (100 MHz, $CDCl_3$) δ 161.08, 158.60, 152.55, 136.93, 136.22, 135.69, 132.25, 132.23, 131.91, 129.10 (2 × CH), 128.68, 128.65, 128.00 (CH), 126.60 (2×CH), 124.14, 123.70, 121.86, 121.43, 48.31 (CH_2Ph), 25.78 (CH_2), 25.53 (CH_2). HR-ESI-MS: 509.11038 $[M+H]^+$ (calc.: 509.11223)

3-benzyl-2-(thiophen-2-yl)-5,6,7,8-tetrahydrobenzo[4,5]thieno[2,3-d]pyrimidin-4(3H)-one (**compound 5**)

Yield 68%, yellow solid; m.p. 185-187 °C; ^{1}H NMR (400 MHz, $CDCl_3$) δ 7.49-7.43 (m, 1H), 7.31 (t, *J* = 7.2 Hz, 2H), 7.28 – 7.23 (m, 1H), 7.17-7.13 (m, 1H), 7.10 (d, J = 7.3 Hz, 2H), 7.01-6.95 (m, 1H), 5.48 (s, 2H), 3.04 (t, *J* = 5.9 Hz, 2H), 2.80 (t, *J* = 5.8 Hz, 2H), 1.95-1.79 (m, 4H). ^{13}C NMR (100 MHz, $CDCl_3$) δ 161.73, 158.88, 149.67, 136.90, 136.68, 134.69, 131.97, 129.54, 129.46, 129.01, 127.51, 127.49, 126.14, 121.03, 48.98 (CH_2Ph), 25.71 (CH_2), 25.50 (CH_2), 23.08 (CH_2), 22.41 (CH_2). HR-ESI-MS: 379.09183 $[M+H]^+$ (calc.: 379.09388)

2-(3-chlorophenyl)-3-(4-hydroxyphenethyl)-5,6,7,8-tetrahydrobenzo[4,5]thieno[2,3-d]pyrimidin-4(3H)-one (**compound 9**)

Yield 56%, yellow solid ;m.p. 238-240 °C; ^{1}H NMR (400 MHz, DMSO-d_6) δ 9.24 (s, 1H), 8.31 (s, 1H), 7.61 (d, *J* = 7.8 Hz, 1H), 7.51 (t, *J* = 7.7 Hz, 1H), 7.41 (d, *J* = 8.0 Hz, 2H), 6.59 (s, 4H), 3.95 (t, *J* = 7.2 Hz, 2H), 2.97 (t, *J* = 6.0 Hz, 2H), 2.78 (t, *J* = 5.4 Hz, 2H), 2.70 (t, *J* = 7.6 Hz, 2H), 1.87-1.76 (m, 4H). ^{13}C NMR (100 MHz, DMSO-d_6) δ 160.50, 157.52, 156.05, 154.12, 136.75, 133.27, 133.07, 130.89, 130.20, 129.63, 129.32 (2×CH), 127.89, 127.75, 126.71, 120.70, 115.30 (2×CH), 47.11 (NCH_2), 32.84

(CH_2Ph), 25.33 (CH_2), 24.62 (CH_2), 22.45 (CH_2), 21.81 (CH_2). HR-ESI-MS: 437.10742 $[M+H]^+$ (calc.: 437.10905)

3-benzyl-2-(furan-2-yl)-3,5,6,7,8,9-hexahydro-4H-cyclohepta[4,5]thieno[2,3-d]pyrimidin-4-one (**compound 11**)

Yield 66%, yellow solid; m.p. 192-194 °C; ^1H NMR (400 MHz, $CDCl_3$) δ 7.55 (s, 1H), 7.29 (d, *J* = 6.9 Hz, 1H), 7.26 – 7.20 (m, 2H), 7.12 (d, *J* = 7.1 Hz, 2H), 6.88 – 6.82 (m, 1H), 6.47 (dd, *J* = 3.3, 1.6 Hz, 1H), 5.57 (s, 2H), 3.36 (t, *J* = 5.5 Hz, 2H), 2.90 (t, *J* = 5.5 Hz, 2H), 1.95 – 1.87 (m, 2H), 1.76-1.64 (m, 4H). ^{13}C NMR (100 MHz, $CDCl_3$) δ 160.22, 159.14, 147.02, 145.43, 144.60 (CH), 139.25, 137.67, 136.93, 128.79 (2×CH), 127.42 (CH), 126.46 (2×CH), 121.85, 115.39 (CH), 112.05 (CH), 47.81 (CH_2Ph), 32.73 (CH_2), 30.24 (CH_2), 27.99 (CH_2), 27.86 (CH_2), 27.39 (CH_2). HR-ESI-MS: 377.13141 $[M+H]^+$ (calc.: 377.13237)

2-(4,5-dimethylthiophen-2-yl)-3-(4-hydroxyphenethyl)-3,5,6,7,8,9-hexahydro-4H-cyclohepta[4,5]thieno[2,3-d]pyrimidin-4-one (**compound 14**)

Yield 74%, yellow solid; m.p. 186-188 °C; ^1H NMR (400 MHz, DMSO-d_6) δ 9.28 (s, 1H), 7.30 (s, 1H), 6.95 (d, *J* = 8.3 Hz, 2H), 6.70 (d, *J* = 8.4 Hz, 2H), 4.30 (t, *J* = 8.0 Hz, 2H), 3.31 (d, *J* = 5.7 Hz, 2H), 2.90 (t, *J* = 9.9 Hz,4H), 2.38 (s, 3H), 2.16 (s, 3H), 1.94 – 1.81 (m, 2H), 1.73 – 1.50 (m, 4H). ^{13}C NMR (100 MHz, DMSO-d_6) δ 159.21, 158.20, 156.02, 148.94, 137.39, 137.33, 136.54, 133.70, 132.35, 130.90, 129.35 (2×CH), 127.89 (CH), 120.24, 115.35 (2×CH), 46.61 (CH_2N), 33.12 (CH_2Ph), 31.97 (CH_2), 29.10 (CH_2), 27.30 (CH_2), 27.04 (CH_2), 26.83 (CH_2), 13.33 (CH_3), 12.73 (CH_3). HR-ESI-MS: 451.14907 $[M+H]^+$ (calc.: 451.15139)

3-benzyl-2-(4-chlorophenyl)-3,5,6,7,8,9-hexahydro-4H-cyclohepta[4,5]thieno[2,3-*d*]pyrimidin-4-one(**compound 16**)

Yield 72%, white solid; m.p. 170-172 °C; ^1H NMR (400 MHz, $CDCl_3$) δ 7.34 (d, *J* = 8.4 Hz, 2H), 7.28-7.20 (m, 5H), 7.01 – 6.90 (m, 2H), 5.23 (s, 1H), 3.39 (t, *J* = 5.4 Hz, 2H), 2.88 (t, *J* = 5.3 Hz, 2H), 1.98-1.88 (m, 2H), 1.80-1.66 (m, 4H). ^{13}C NMR (100 MHz, $CDCl_3$) δ 180.05 (NCO), 159.26, 154.43, 138.98, 137.60, 136.76, 136.29, 133.61, 129.70 (2×CH), 128.90 (2×CH), 128.83 (2×CH), 127.61 (CHPh), 126.71 (2×CH), 122.04, 48.59 (CH_2Ph), 32.75 (CH_2), 30.21 (CH_2), 28.02 (CH_2), 27.90 (CH_2), 27.45 (CH_2). HR-ESI-MS: 421.11175 $[M+H]^+$ (calc.: 421.11414)

3-benzyl-2-(4-fluorophenyl)-3,5,6,7,8,9-hexahydro-4H-cyclohepta[4,5]thieno[2,3-d]pyrimidin-4-one (**compound 17**)

Yield 61%,white solid; m.p. 146-148 °C;^1H NMR (400 MHz, $CDCl_3$) δ 7.34 – 7.19 (m, 5H), 7.05 (t, *J* = 8.5 Hz, 2H), 6.99 – 6.89 (m, 1H), 5.24 (s, 1H), 3.47 – 3.34 (m, 1H), 2.94 – 2.83 (m, 1H), 1.98 – 1.88 (m,

1H), 1.73 (tt, *J* = 11.4, 5.9 Hz, 2H). ^{13}C NMR (100 MHz, $CDCl_3$) δ 166.55 (NCO), 158.74, 154.44, 154.44, 140.83, 140.14, 136.63, 136.37, 133.47, 129.77, 128.92 (2×CH), 128.83 (2×CH), 127.68 (CH), 126.83 (2×CH), 119.14, 48.63 (CH_2Ph), 29.86 (CH_2), 29.15 (CH_2), 28.23 (CH_2). HR-ESI-MS: 405.14217 $[M+H]^+$ (calc.: 405.14369)

3-benzyl-5,6-dimethyl-2-(thiophen-2-yl)thieno[2,3-d]pyrimidin-4(3H)-one (**compound 20**)

Yield 71%, white solid; m.p. 162-164 °C; ^{1}H NMR (400 MHz, $CDCl_3$) δ 7.46 (d, *J* = 5.1 Hz, 1H), 7.32 (t, *J* = 7.3 Hz, 2H), 7.29 – 7.23 (m, 1H), 7.15 (d, *J* = 3.7 Hz, 1H), 7.10 (d, *J* = 7.3 Hz, 2H), 7.01-6.93 (m, 1H), 5.48 (s, 2H), 2.50 (s, 3H), 2.42 (s, 3H). ^{13}C NMR (100 MHz, $CDCl_3$) δ 161.01, 159.11 (CO), 149.63, 136.91, 136.65, 131.46, 129.92, 129.51 (CH), 129.44 (CH), 129.03 (2×CH), 127.51 (CH), 127.49 (CH), 126.07 (2×CH), 121.82, 49.06 (CH_2Ph), 13.36 (CH_3), 13.23 (CH_3).

3-(4-hydroxyphenethyl)-5,6-dimethyl-2-phenylthieno[2,3-d]pyrimidin-4(3H)-one (**compound 22**)

Yield 66%, white solid; m.p. 251-253 °C; ^{1}H NMR (400 MHz, DMSO-d_6) δ 9.23 (s, 1H), 7.62 – 7.47 (m, 5H), 6.60 (dd, *J* = 11.7 Hz, 4H), 3.98 (t, *J* = 7.7 Hz, 2H), 2.70 (t, J = 8.1 Hz, 2H), 2.48 (s, 3H), 2.41 (s, 3H). ^{13}C NMR (100 MHz, DMSO-d_6) δ 160.00, 157.87, 155.94, 155.47, 134.97, 129.99, 129.69 (CH), 129.22 (2×CH), 128.75, 128.32 (2×CH), 127.98 (2×CH), 127.83, 121.27, 115.24 (2×CH), 46.92 (NCH_2), 33.03 (CH_2Ph), 12.90 (CH_3), 12.76 (CH_3). HR-ESI-MS: 377.12927 $[M+H]^+$ (calc.: 377.13237)

NMR spectrum of compounds **4**, **5**, **9**, **11**, **14**, **16**, **17**, **20**, and **22**

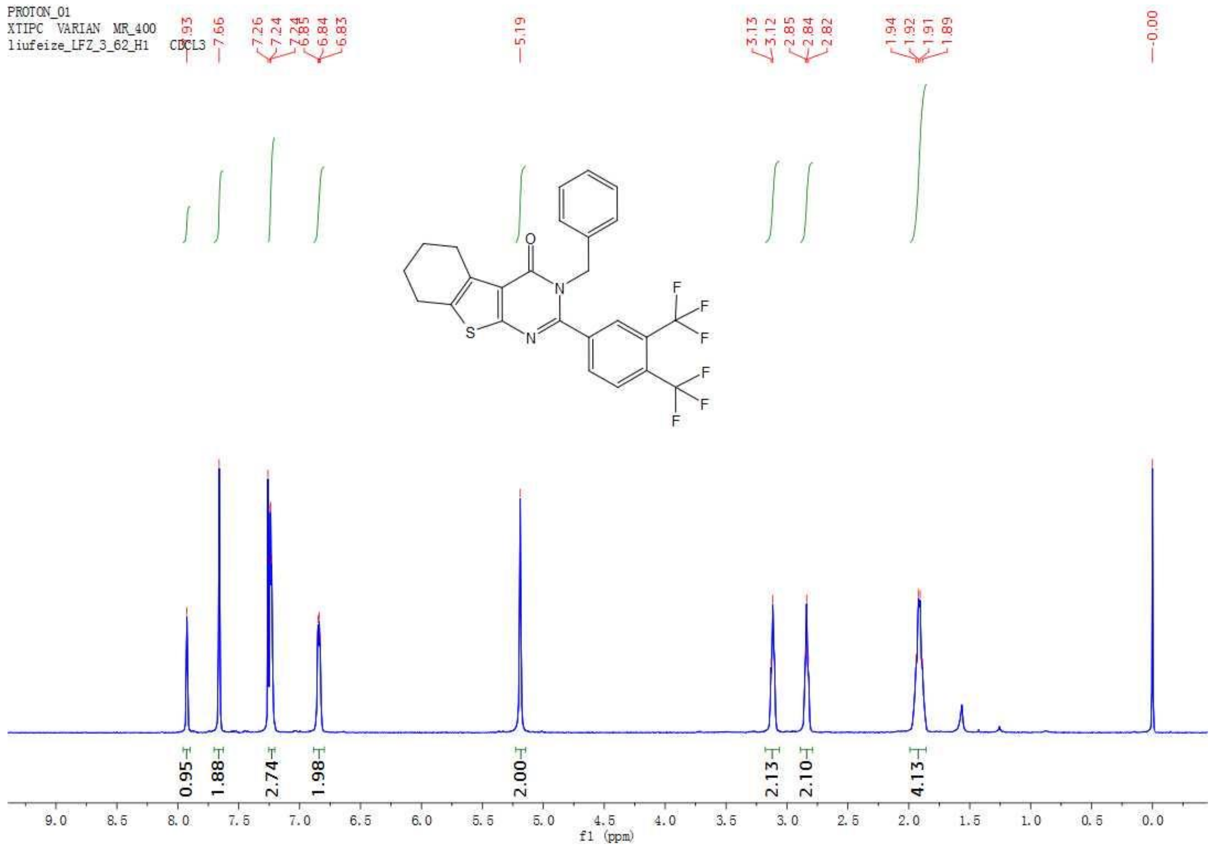

[1] H NMR of **compound 4** ($CDCl_3$, 400 MHz)

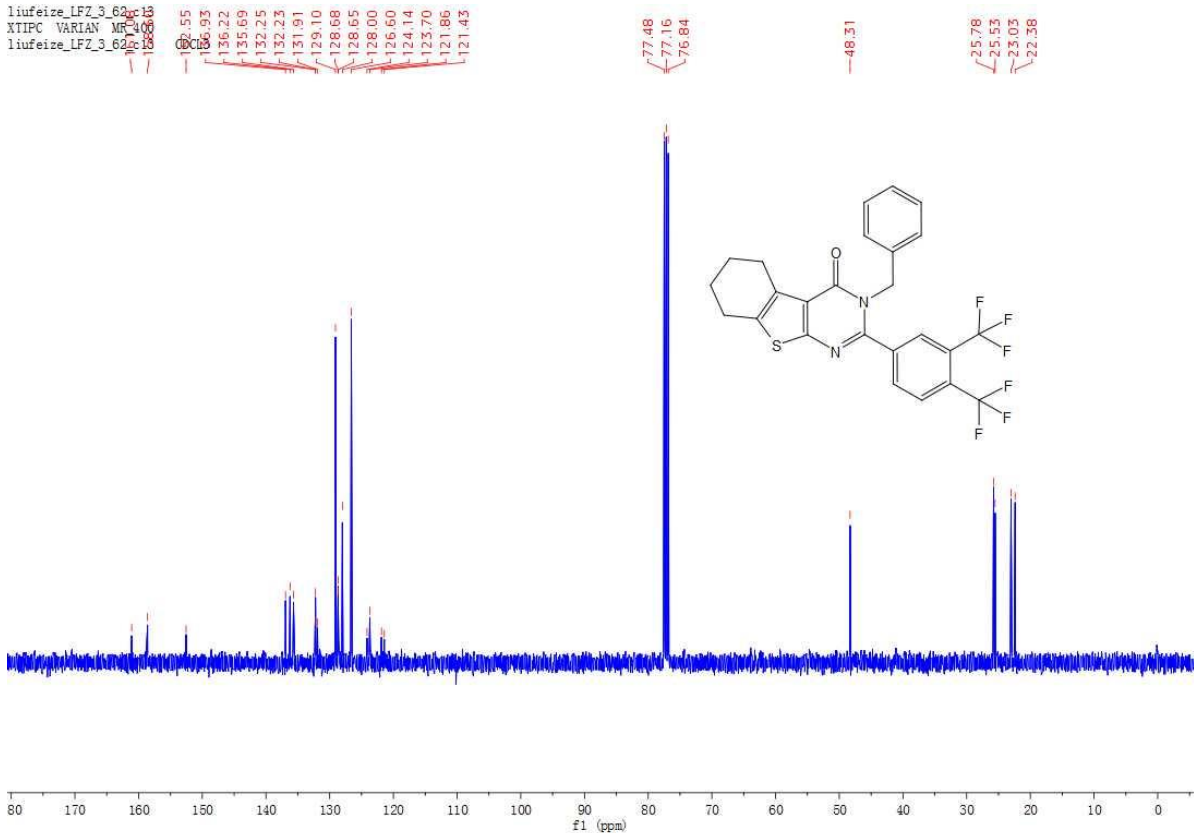

^{13}C NMR of **compound 4** ($CDCl_3$, 100 MHz)

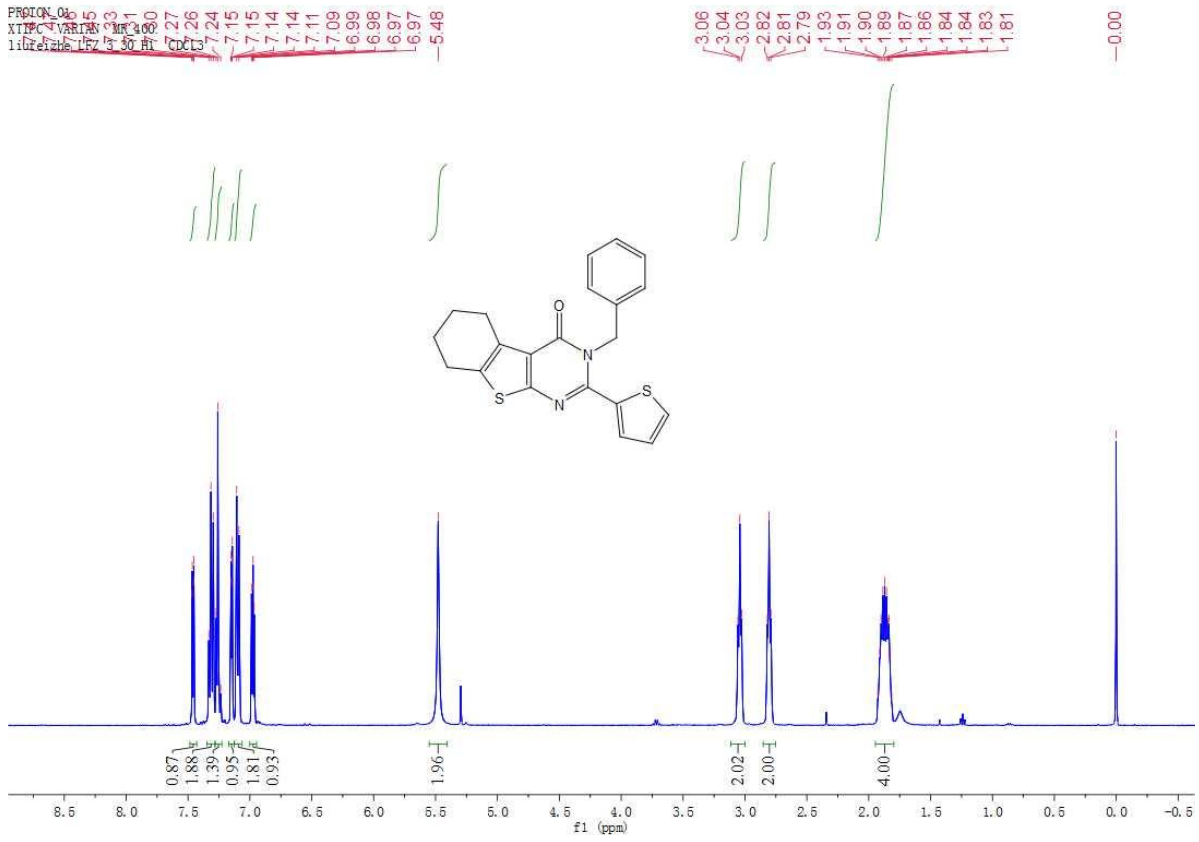

[1] H NMR of **compound 5** ($CDCl_3$, 400 MHz)

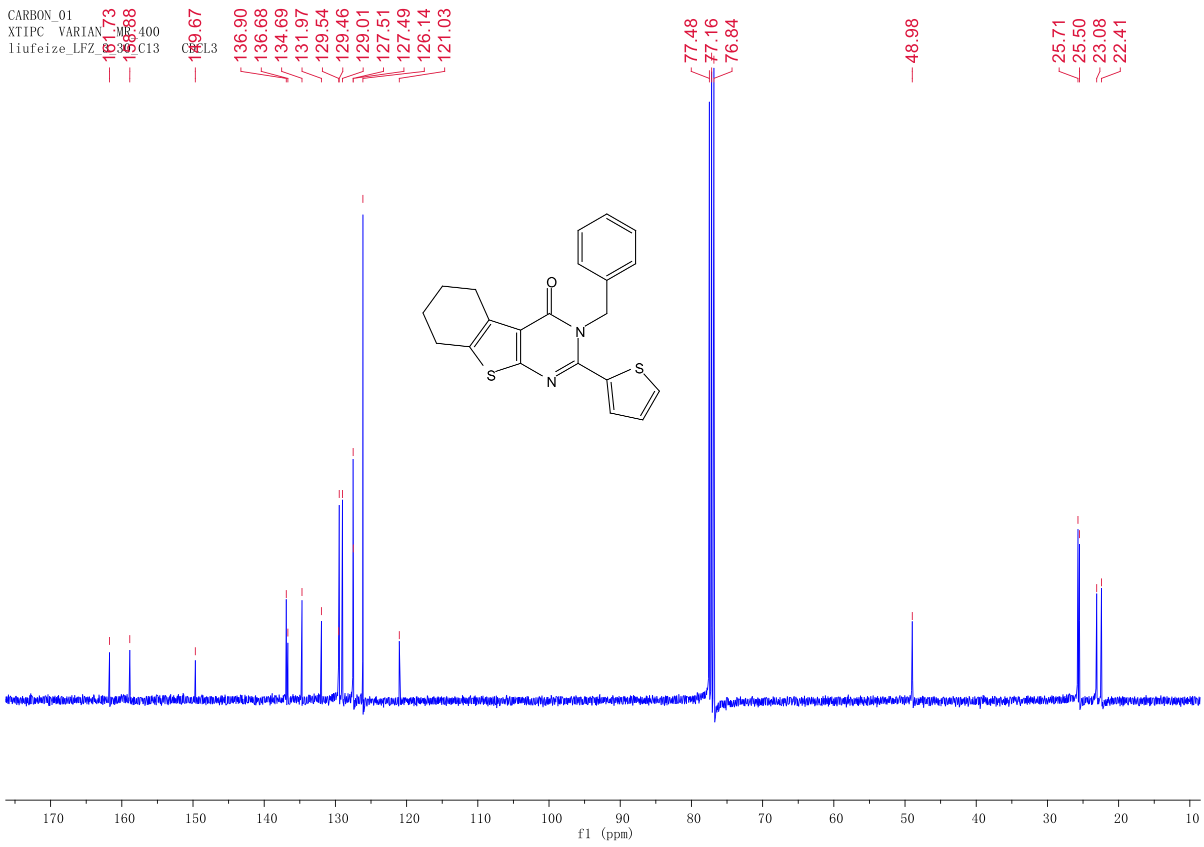

^{13}C NMR of **compound 5** ($CDCl_3$, 100 MHz)

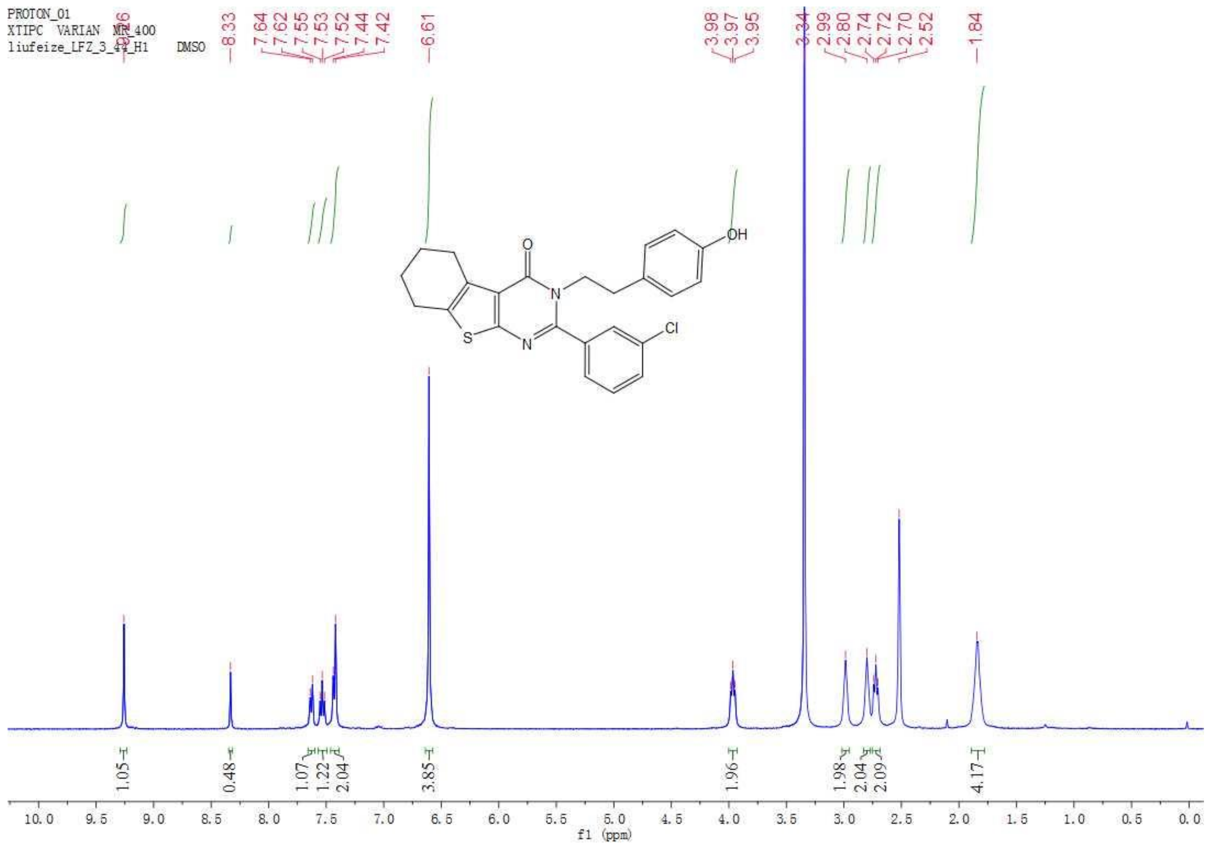

[1] H NMR of **compound 9** (DMSO-d_6, 400 MHz)

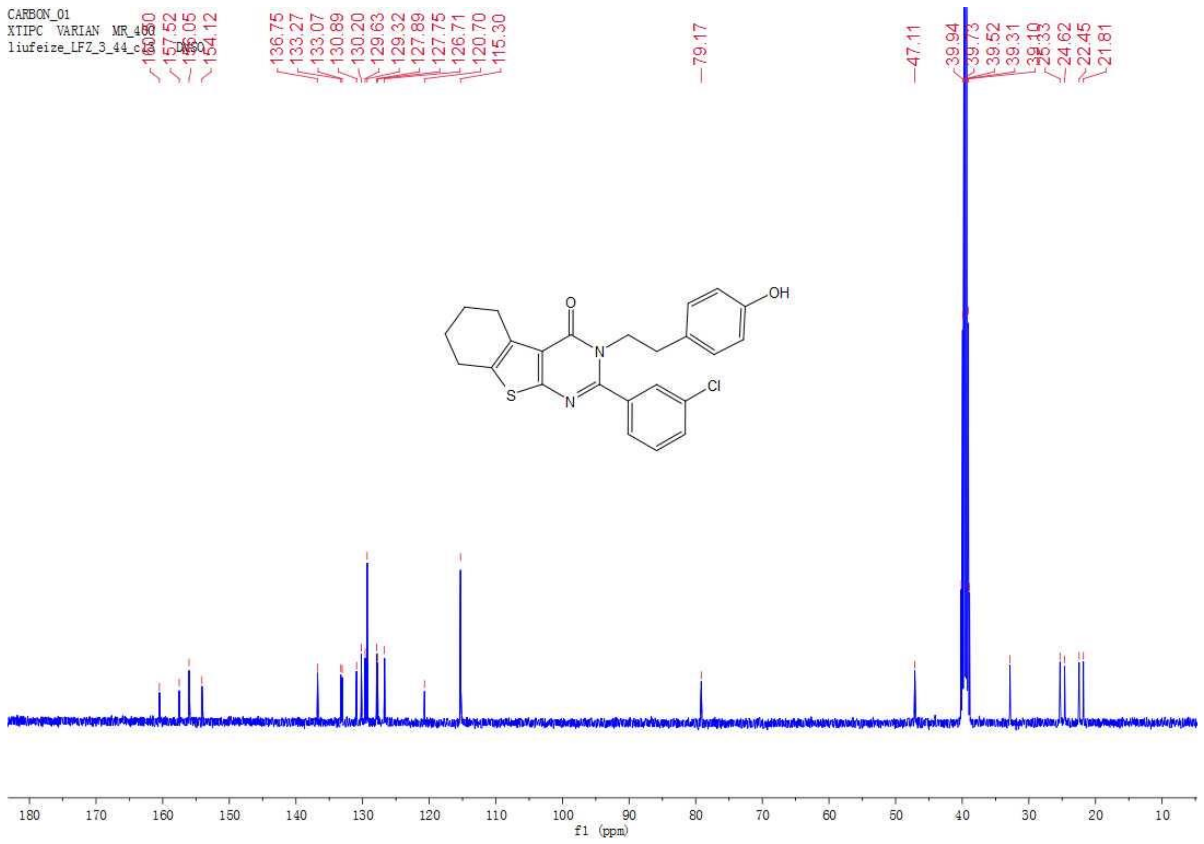

^{13}C NMR of **compound 9** (DMSO-d_6, 100 MHz)

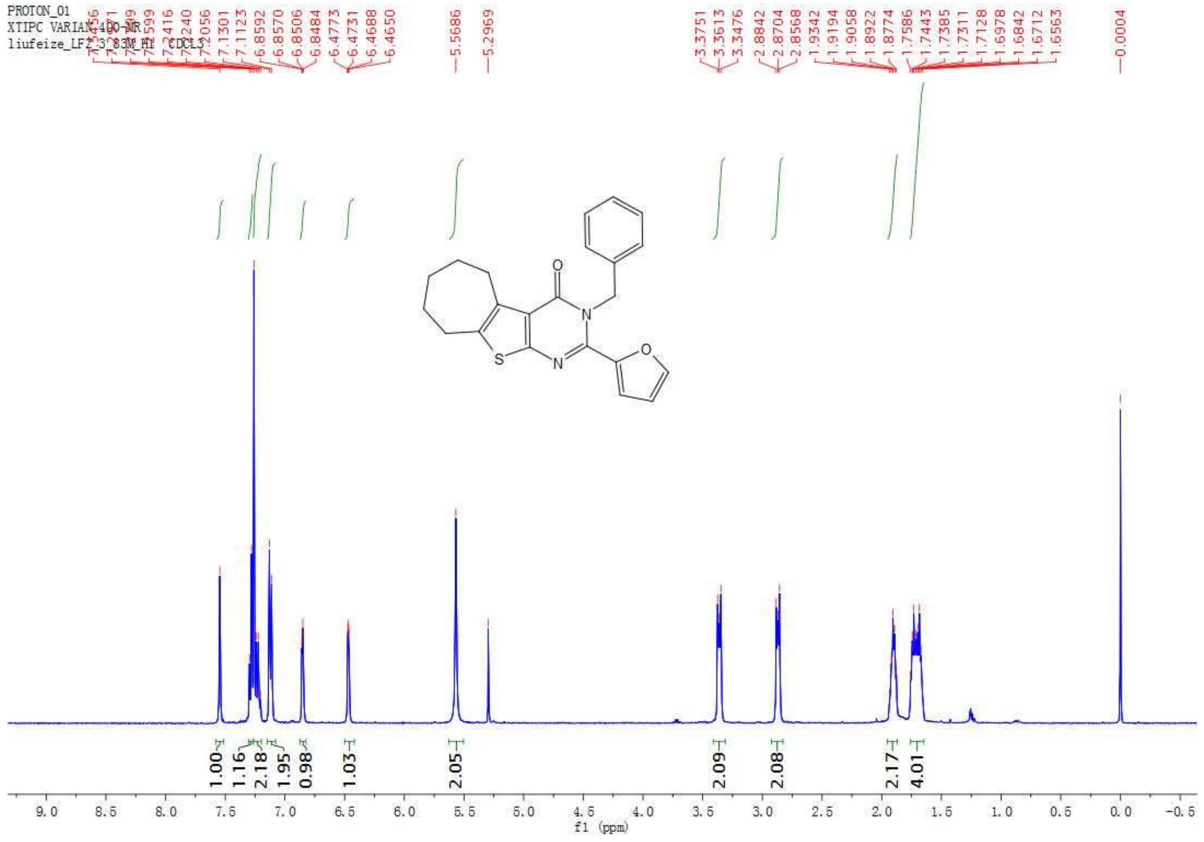

[1] H NMR of **compound 11** ($CDCl_3$, 400 MHz)

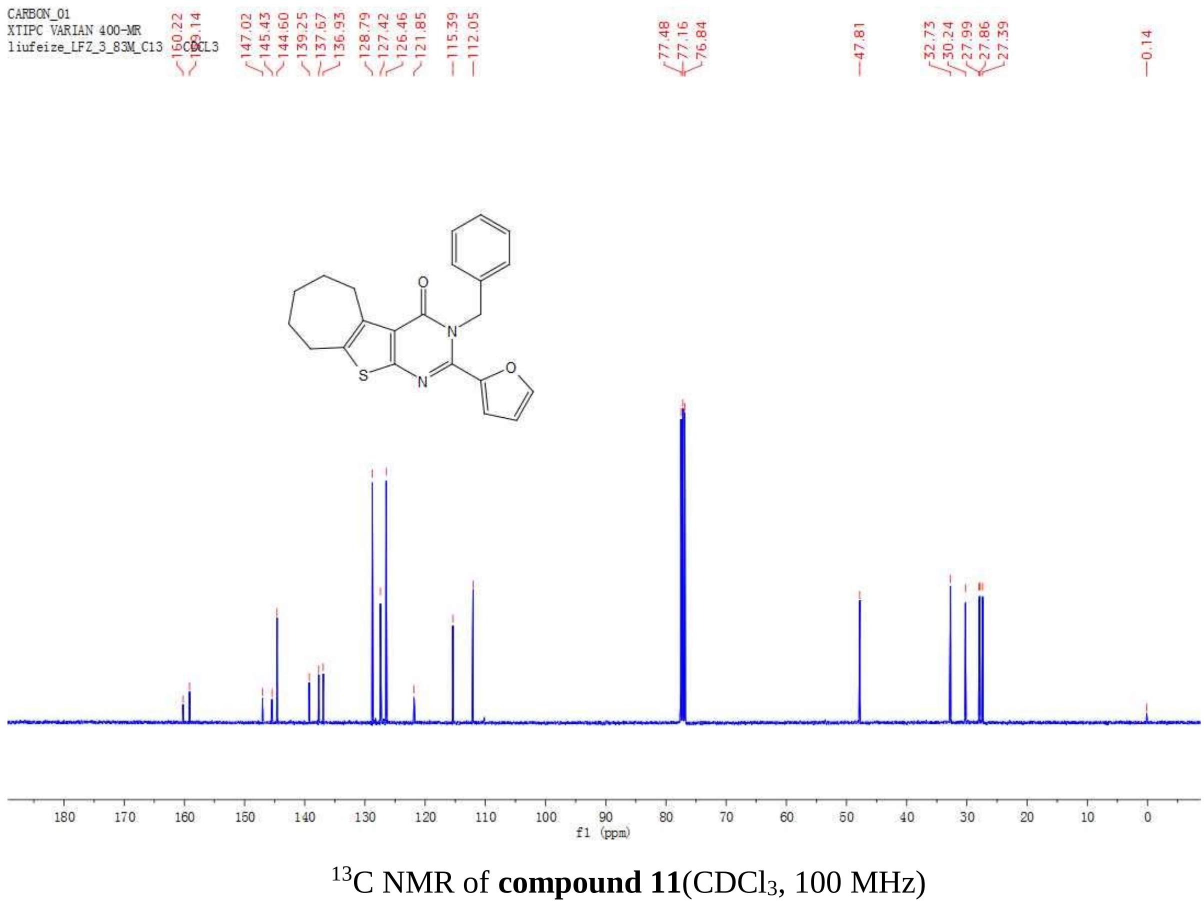

^{13}C NMR of **compound 11**($CDCl_3$, 100 MHz)

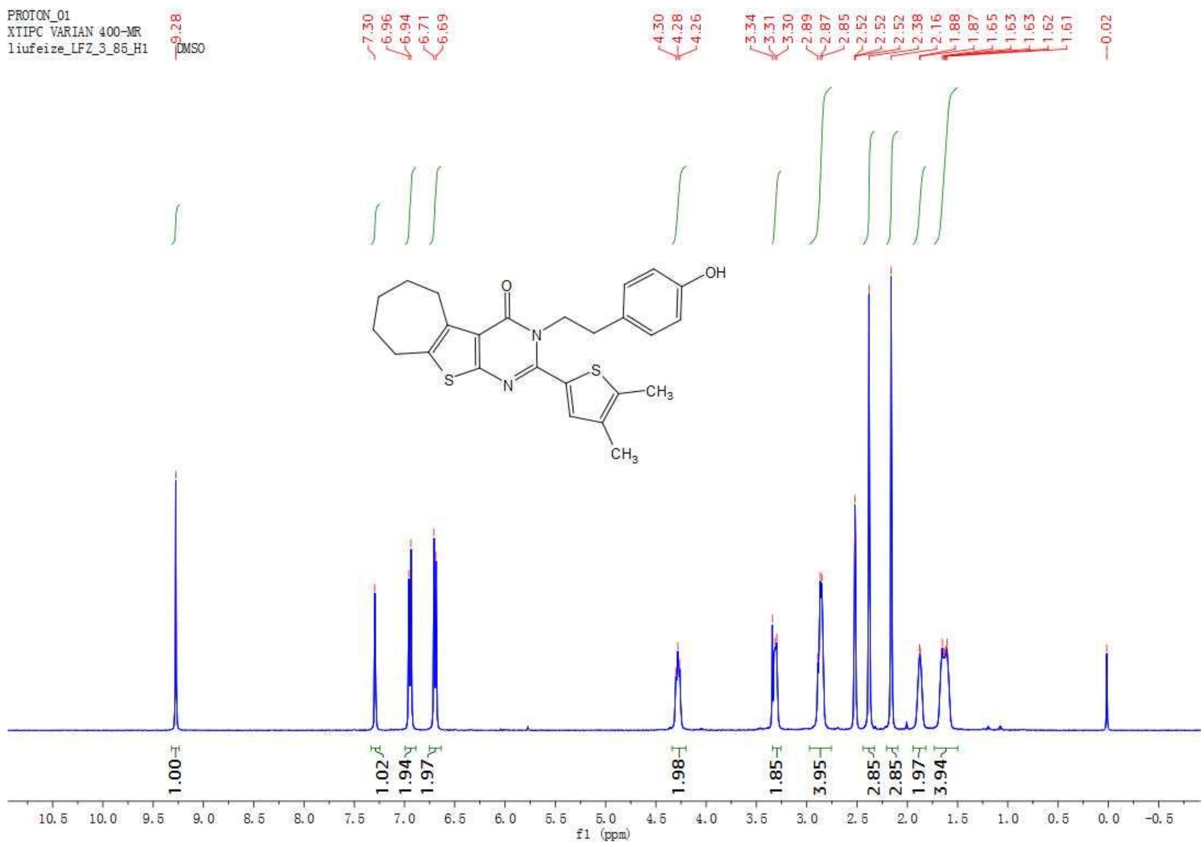

[1] H NMR of **compound 14** (DMSO-d_6, 400 MHz)

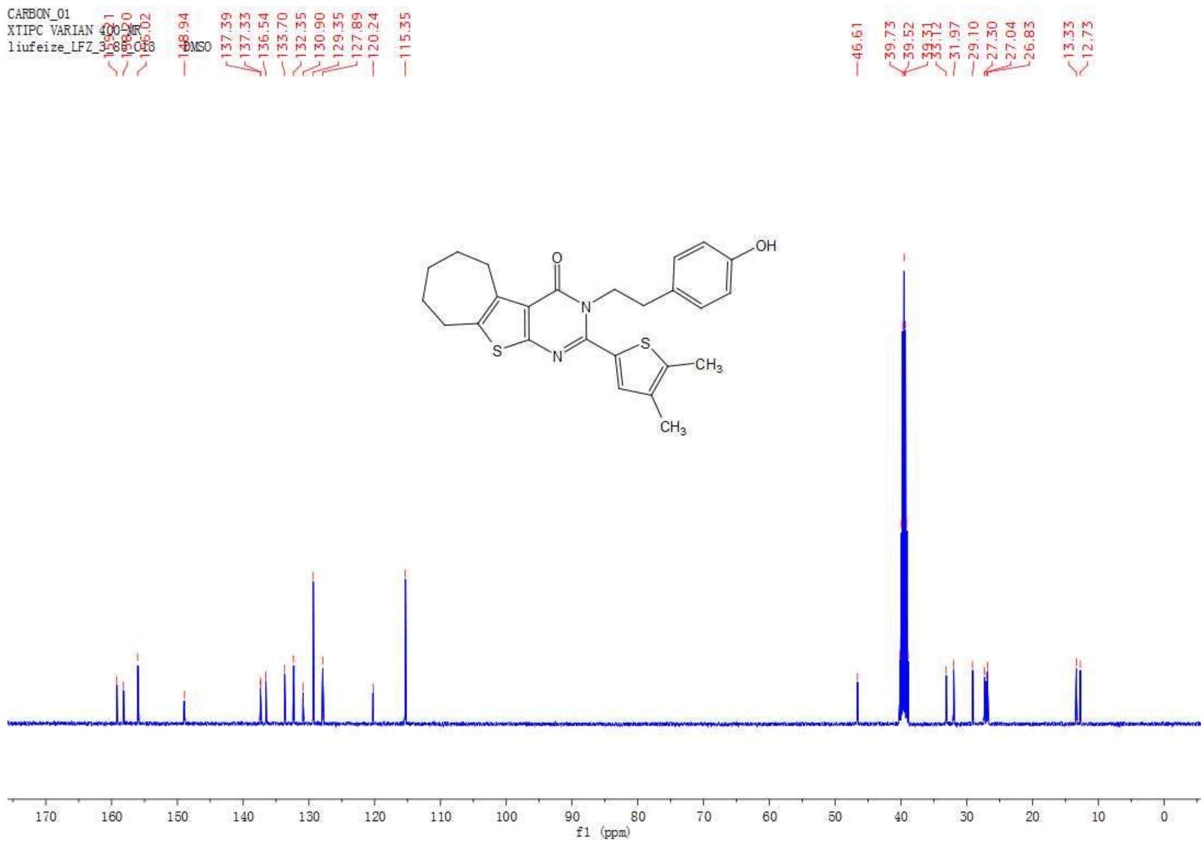

^{13}C NMR of **compound 14** (DMSO-d_6, 100 MHz)

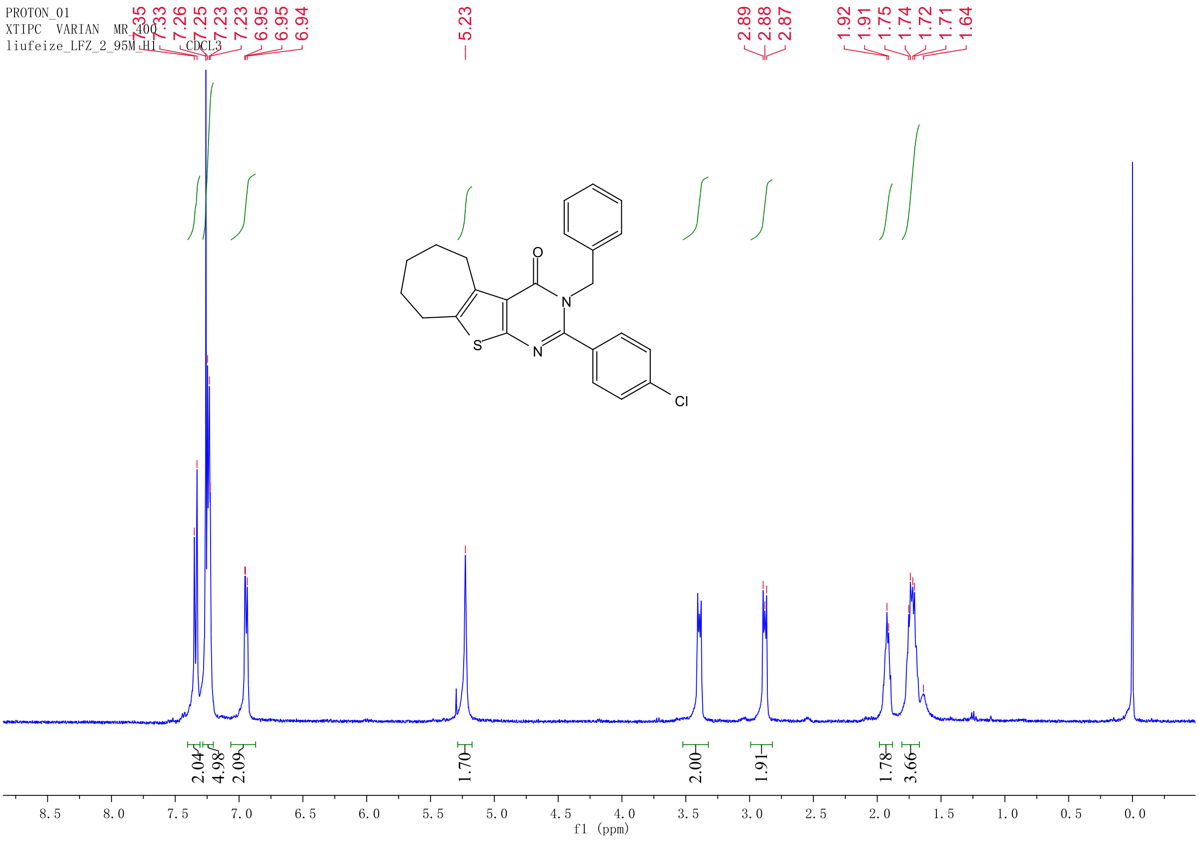

[1] H NMR of **compound 16** ($CDCl_3$, 400 MHz)

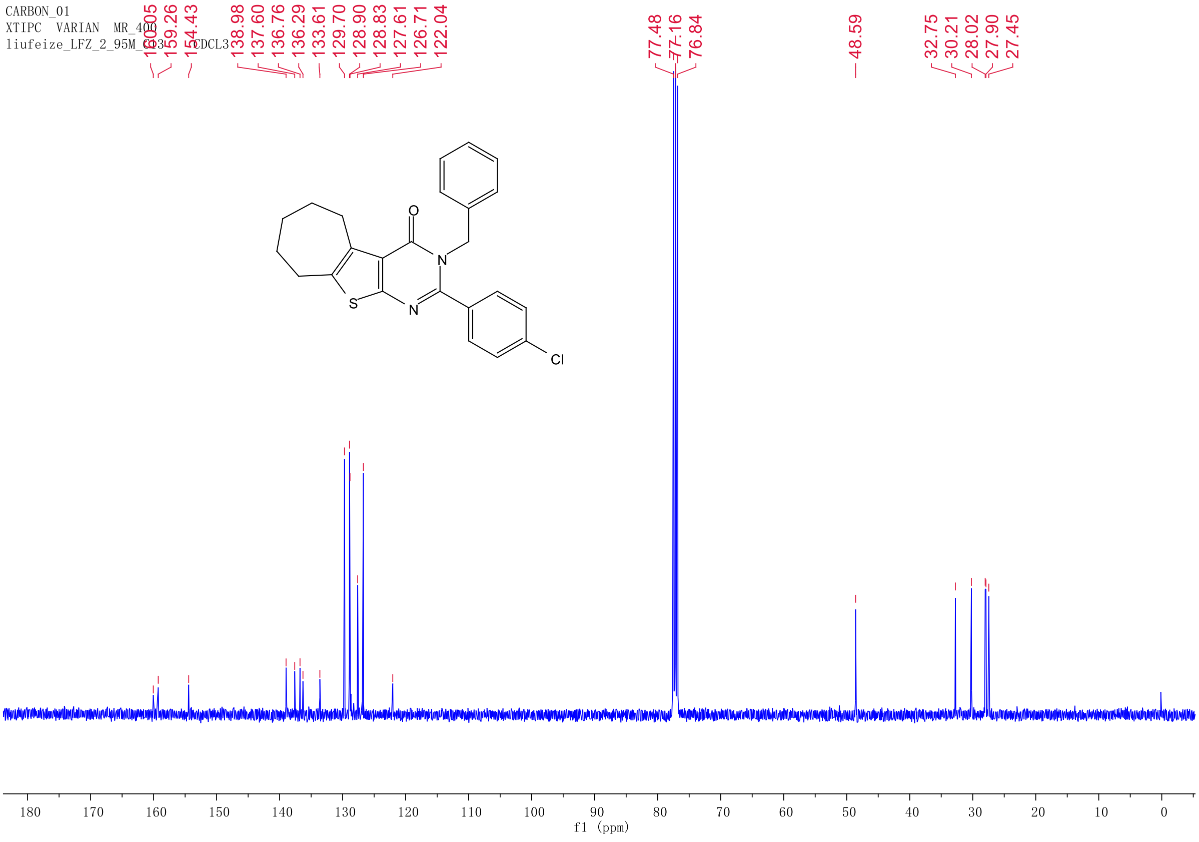

^{13}C NMR of **compound 16** ($CDCl_3$, 100 MHz)

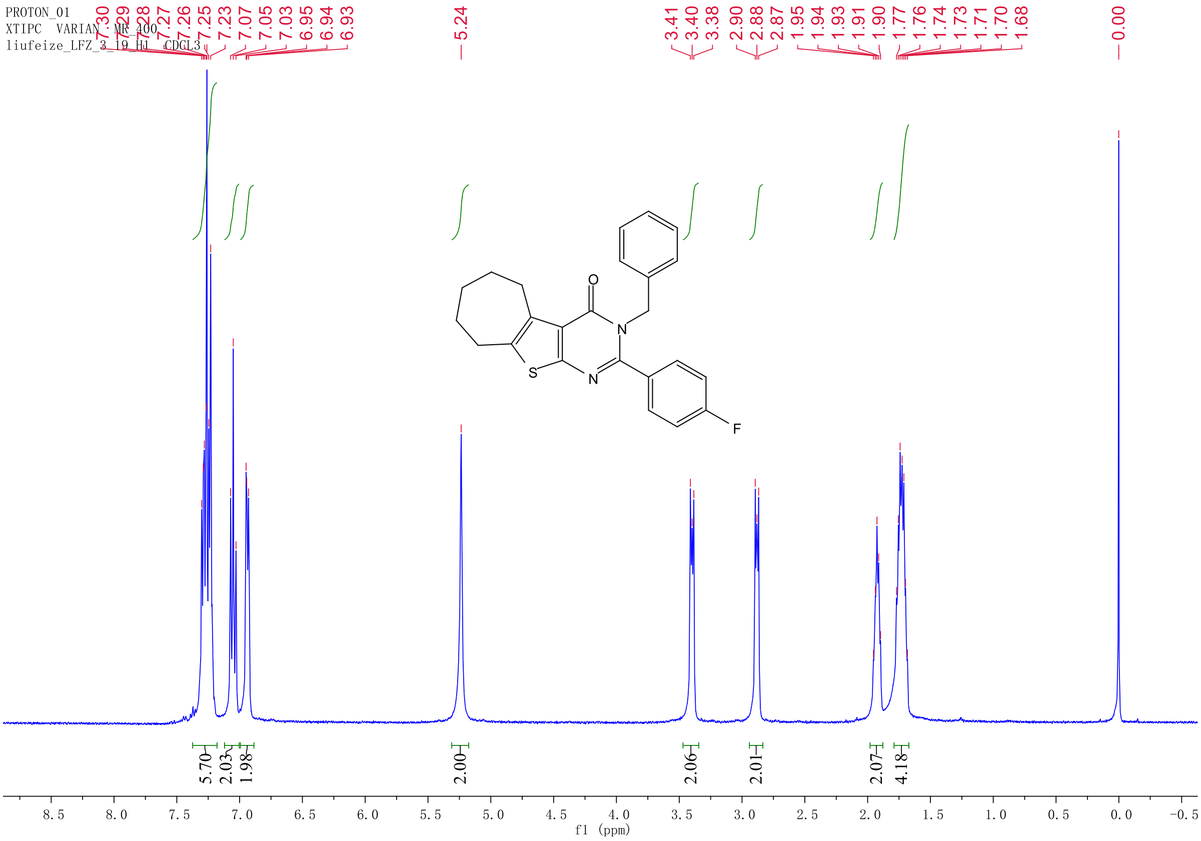

[1] H NMR of **compound 17** ($CDCl_3$, 400 MHz)

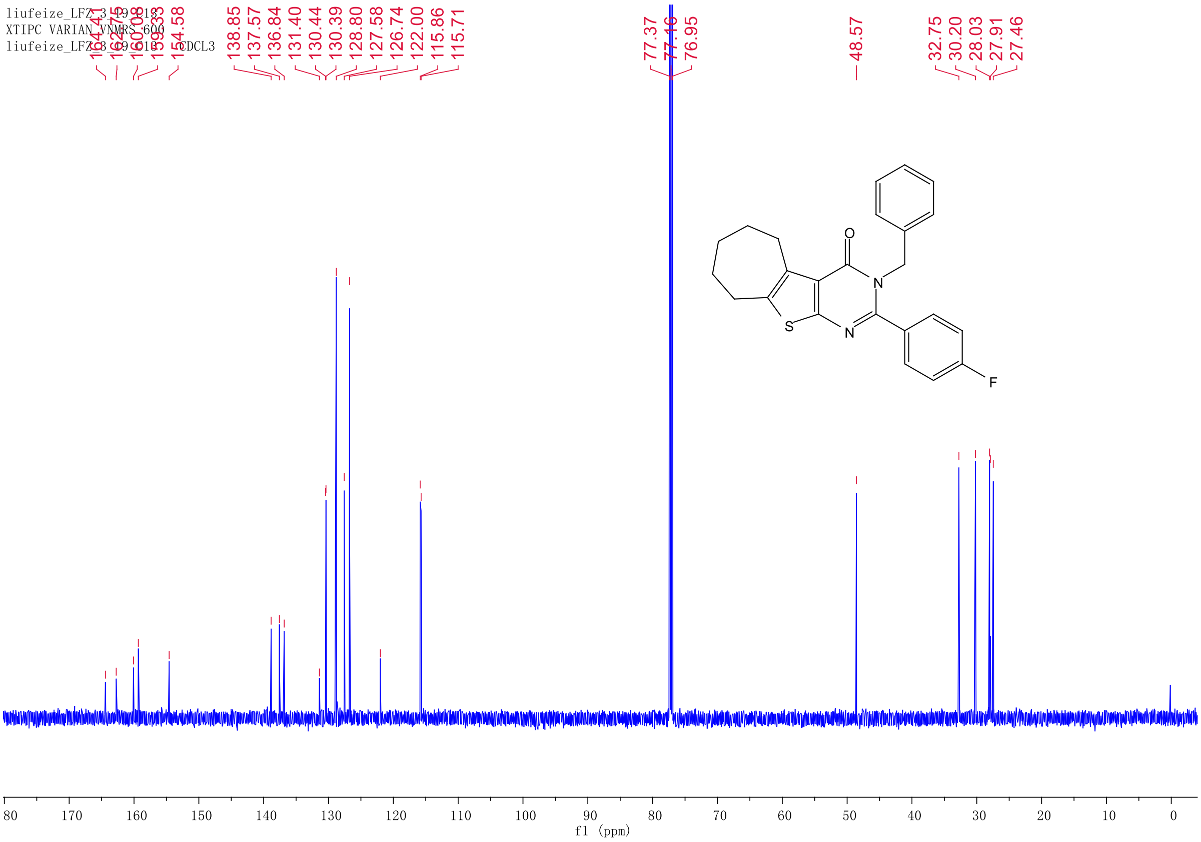

^{13}C NMR of **compound 17** ($CDCl_3$, 100 MHz)

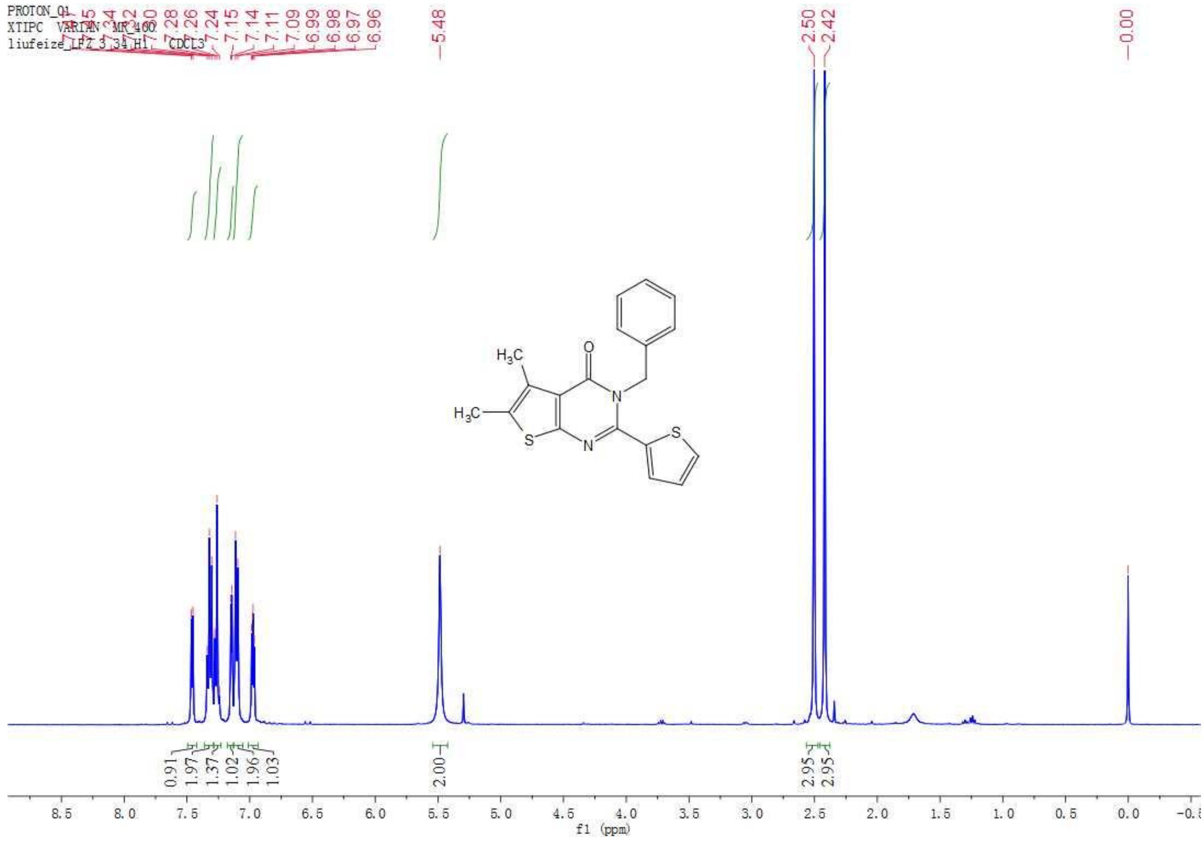

[1] H NMR of **compound 20** ($CDCl_3$, 400 MHz)

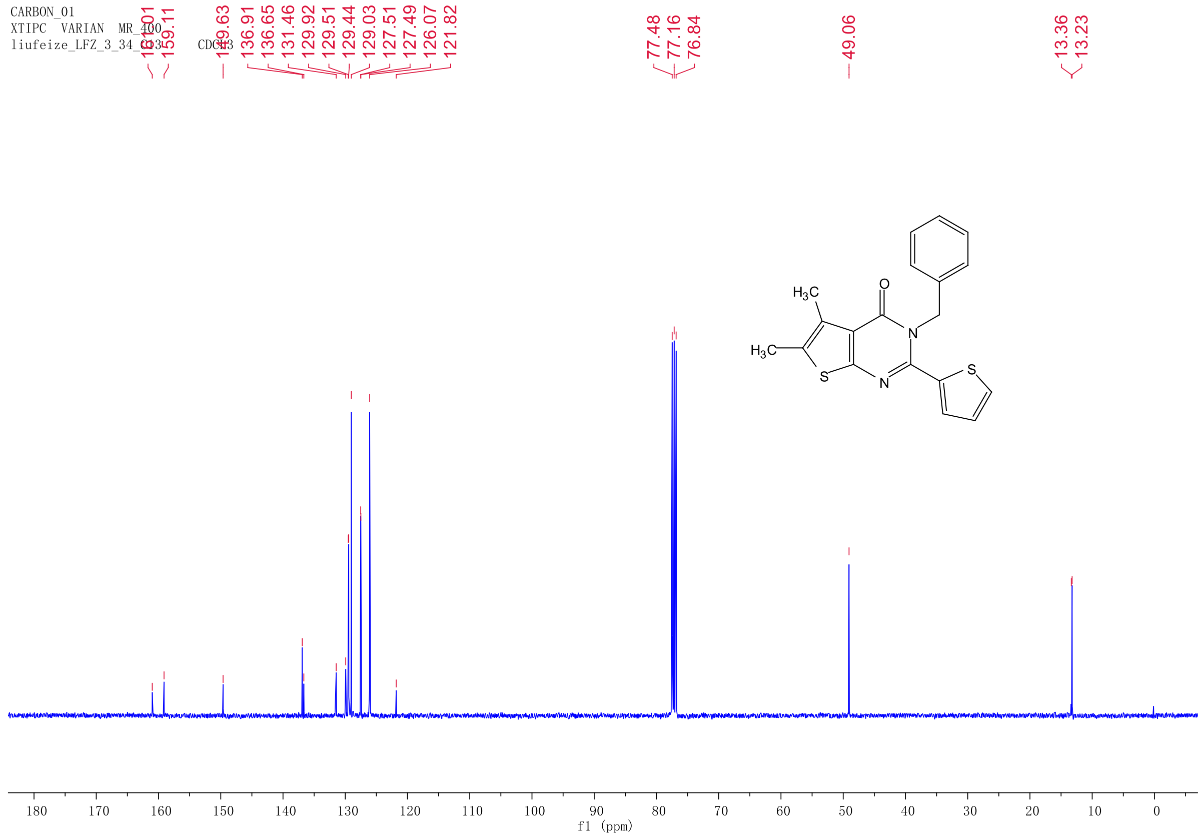

^{13}C NMR of **compound 20** ($CDCl_3$, 100 MHz)

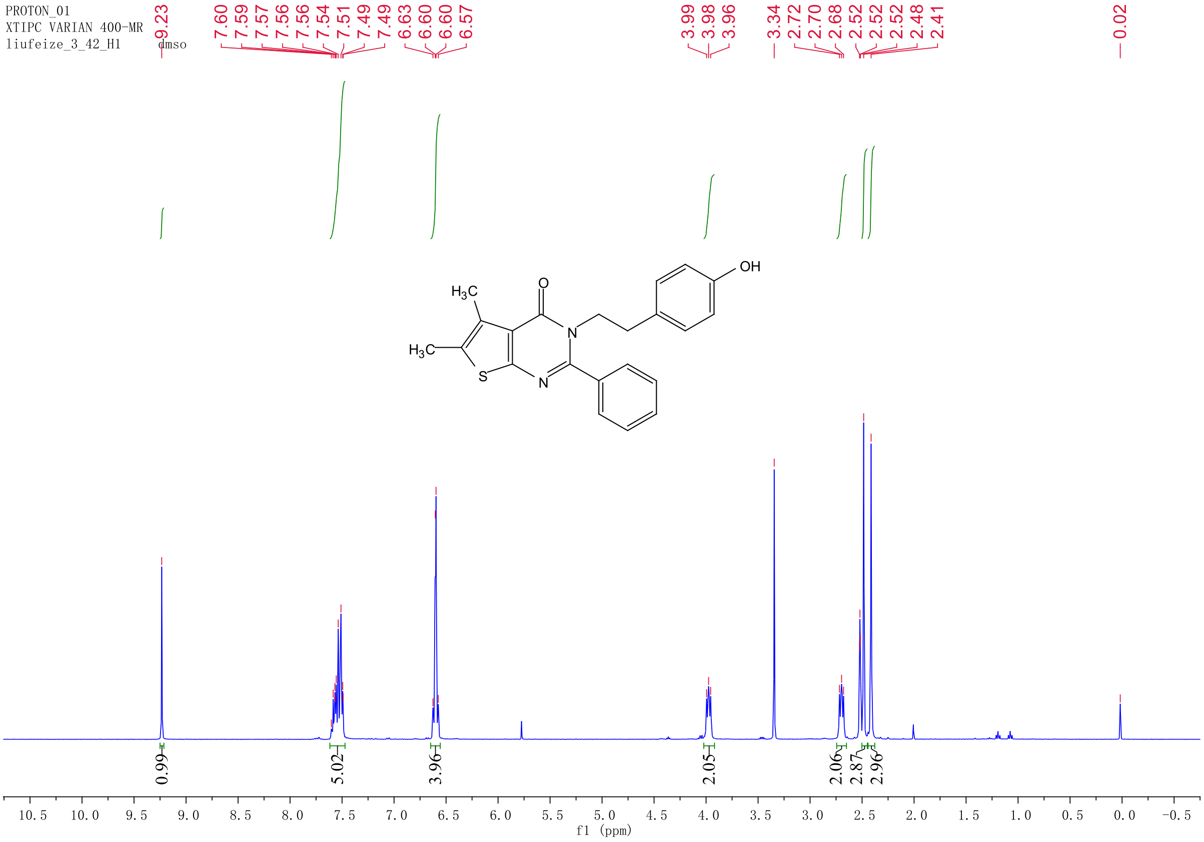

[1] H NMR of **compound 22** (DMSO-d_6), 400 MHz)

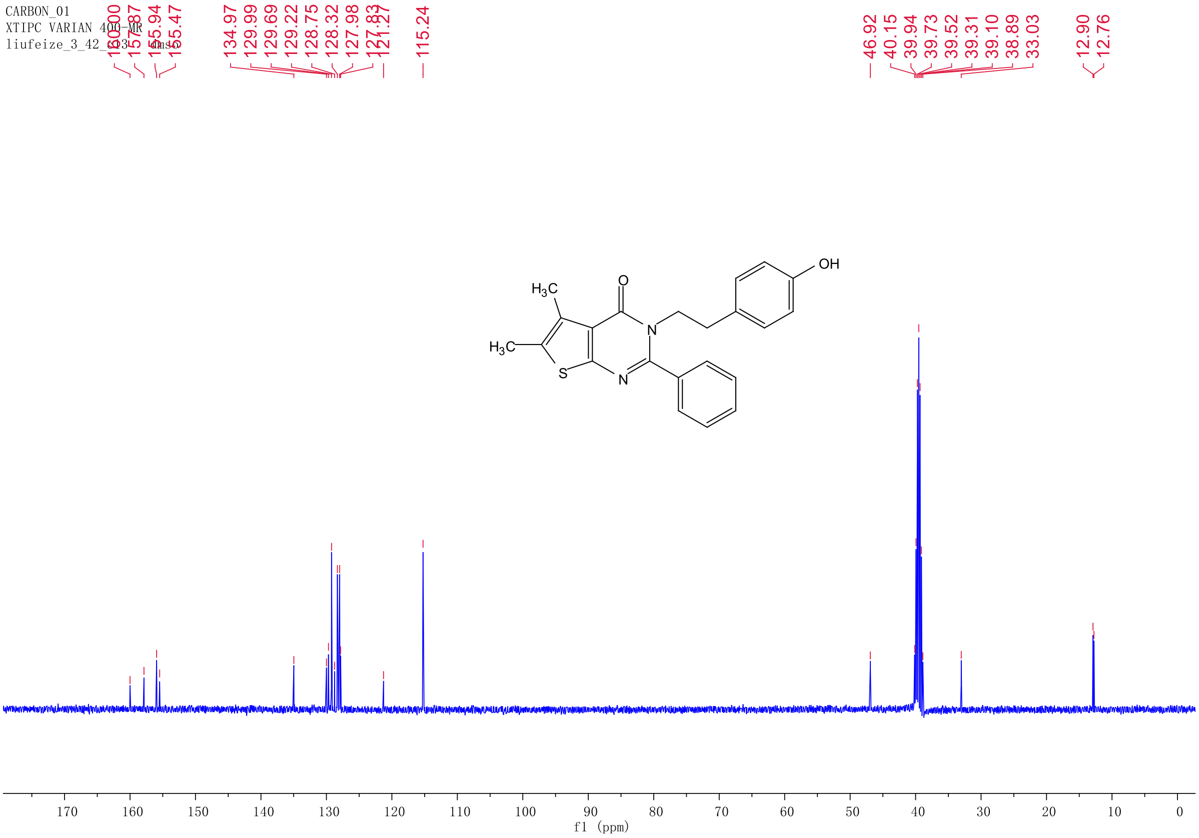

^{13}C NMR of **compound 22** (DMSO-d_6, 100 MHz)
